# Electrochemical impedance spectroscopy for characterizing neural electrodes

**DOI:** 10.1016/j.coelec.2025.101807

**Published:** 2026-01-28

**Authors:** Cynthia C. Eluagu, Bernard W. Biney, Stuart F. Cogan, Kevin J. Otto, Mark E. Orazem

**Affiliations:** 1 Department of Chemical Engineering, University of Florida, Gainesville, FL 32611, USA; 2 Department of Bioengineering, University of Texas at Dallas, Richardson, TX 75080-3021, USA; 3 Weldon School of Biomedical Engineering, Purdue University, West Lafayette, IN 47907-2032, USA

## Abstract

Electrochemical impedance spectroscopy (EIS) has been extensively employed in the field of neural stimulation over the past 25 years. This review summarizes the early applications, major contributions, rudimentary use, and recent advances of EIS in neural applications. EIS is widely used in both research and clinical neurostimulation to monitor changes in electrode impedance due to foreign body response and glial encapsulation. The key parameters for in vitro and in vivo measurements are discussed along with the guidelines for data interpretation.

## Introduction

Electrochemical impedance spectroscopy (EIS) is a non-invasive technique for characterizing the properties of materials across a wide frequency range. By applying small-amplitude sinusoidal potential or current and measuring the response, EIS provides information about electrochemical reactions, system capacitance, mass transfer, and other interfacial phenomena.

Impedance measurements may employ two electrodes, in which the potential of a working electrode is measured against a combined counter and reference electrode. Alternatively, three electrodes may be used with separate counter and reference electrodes. The primary considerations are that the system under investigation is stationary and that the impedance measured falls within the region bounded by the resistance and inductance of the leads (low impedance limit) and the capacitance of the leads (high impedance limit). Use of EIS over a broad frequency range enables evaluation of interface properties unknowable from single-frequency characterization. In a biotic—abiotic interface, this information is useful for separating the influence of implant properties from that of tissue responses after implantation. Early neural-stimulation researchers applied EIS to evaluate electrode—tissue interfaces and establish safe charge-injection limits for device performance and electrical stimulation. In 1962, Adey et al. [1] performed impedance measurements in the brains of cats using chronically implanted coaxial electrodes to investigate the electrophysiological properties of brain tissue. In 1964, Porter et al. [2] measured the impedance of the human brain using stainless-steel tubing and a Wheatstone bridge. Notable work by Rose and Robblee [3] established safe charge-injection limits for platinum electrodes. In 1990, McCreery et al. [4] identified combinations of charge density and charge per phase as damaging and non-damaging.

In 1992, Shannon [5] developed safe levels for electrical stimulation based on current flow principles and damage mechanisms in macroelectrodes, following the work of McCreery et al. [4] The safe charge-injection limit is the maximum charge deliverable per phase (*μ*C/cm^2^) that does not cause tissue damage or harmful faradaic reactions such as water electrolysis. Polarization of electrodes beyond these limits increases the risk of electrode degradation, glial scar formation, and high impedance, which reduces the efficacy of stimulation and recording. Grill and Mortimer [6] demonstrated that encapsulation tissues significantly increased the resistivity and impedance of the surrounding tissues and implants. Faradaic and non-faradaic mechanisms underlying electrode and tissue damage govern the requirement for safe charge delivery during neural stimulation.

Electrophysiologically, there is no possible way to discriminate strong but distant (from the electrode) neural signals from weak but closer sources. The extracellular matrix and pericellular spaces are the dominant materials for transmission through the brain. Both of these are primarily (if not completely) resistive. Thus, there is no mechanism for phase shifting due to distance traveled. Rather, the signals are merely linearly attenuated proportional to their distance. Neural stimulation studies have reported that an increase in impedance over time is attributed to the formation of fibrous encapsulation tissue around implanted electrode arrays. Several strategies have been developed to improve neural implant longevity, reduce tissue encapsulation, and maintain low impedance. Electrode miniaturization to ultra-small dimensions (<10 μm) [7] decreases acute tissue damage during insertion and reduces inflammatory response. Ultramicroelectrodes and microelectrodes with diameters ranging from 5 μm to 50 μm are designed to enhance spatial selectivity for stimulation and recording [8]. Common neural electrode materials such as gold [9], platinum [10], SIROF [11], titanium nitride (TiN) [12], sputtered ruthenium oxide (RuO_x_) [13], and PEDOT:PSS [14] were selected for high charge-injection and low impedance. Cogan et al. [15] showed that SIROF coatings demonstrate high charge-injection capacity with increasing film thickness. A review on electrode materials can be found elsewhere [16].

Rejuvenation offers an alternative solution for reducing electrode impedance. Controlled voltage biases are applied to electrodes to reverse some tissue encapsulation effects and restore signal quality without requiring implant replacement. Otto et al. [17] showed that large voltage pulses of 4 s applied to chronically implanted neural microelectrodes doubled the signal-to-noise ratio and reduced the 1-kHz impedance by over 60 %. These pulses lower the resistive influence of tissue components at the interface. O’Sullivan et al. [18] have recently reported a decrease in impedance response associated with rejuvenation, consistent with previous work by Johnson et al. [19] and Otto et al. [17]. Studies have demonstrated that small voltage pulses and potential-biased asymmetric waveforms up to 1 ms [20] can enhance the charge-injection capacity of neural electrodes.

## Electrochemical impedance spectroscopy measurements

**Figure 1:**
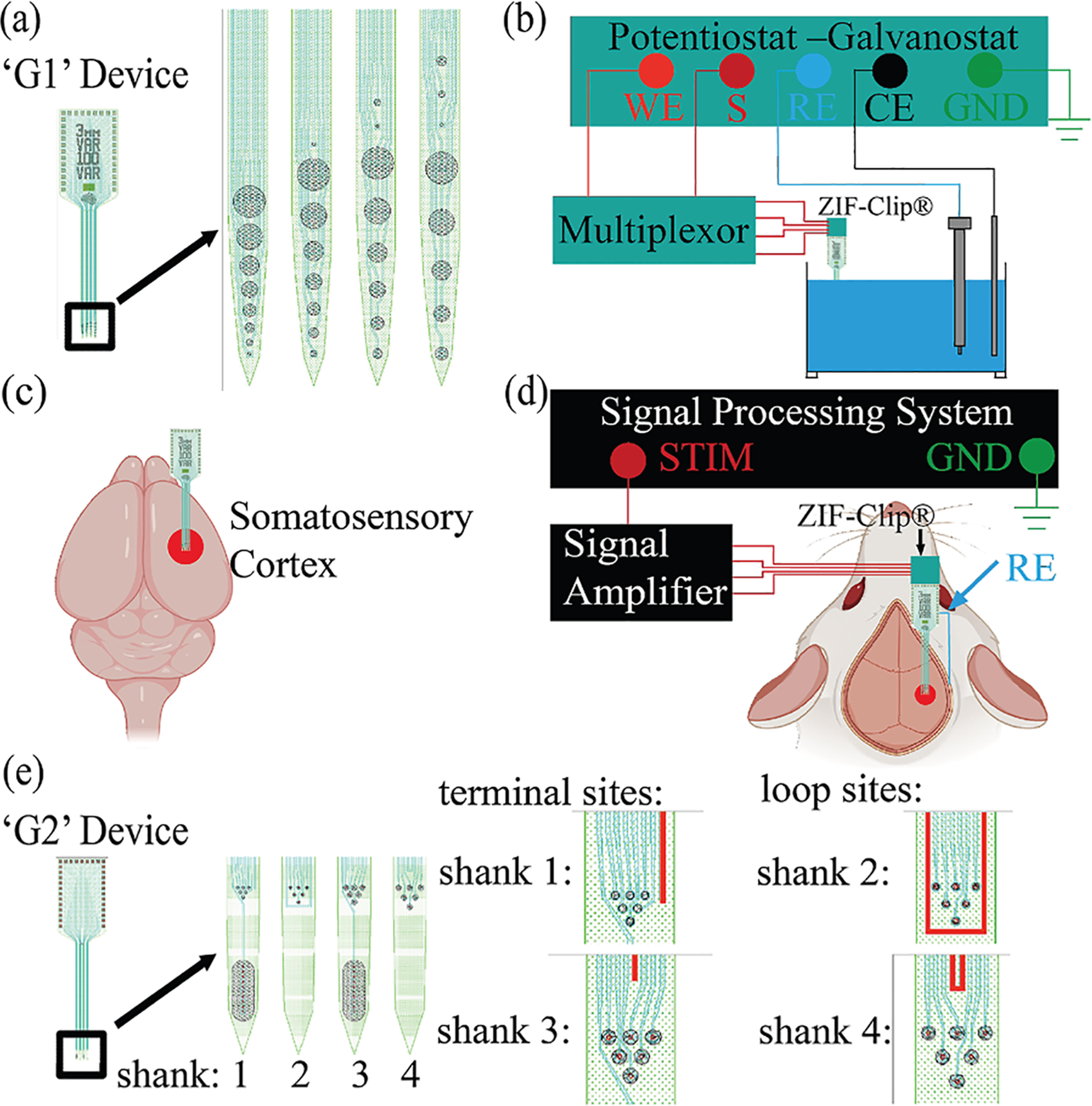
A schematic representation of the experimental setup for electrochemical impedance spectroscopy and neural stimulation, taken from Dong et al. [21]: (**a**) G1 device, consisting of a silicon-based substrate with 4 shanks and 32 sites, ranging from 5 μm to 50 μm in diameter; (**b**) in vitro three-electrode EIS setup where the working/working sense are connected to a multiplexer for channel selection; (**c**) somatosensory cortex of a rat brain, the target area for neural interfacing; (**d**) signal processing system setup for in vivo EIS, including the stimulator connected to the signal amplifier and the ground (GND) reference, the ZIF-ClipⓇ headstage connected to the implanted UMEA with an additional RE placed on the skin; and (e) G2 device, silicon-based substrate with 4 shanks and 24-ultramicroelectrode sites of 5 μm or 10 μm diameter. The terminals and loops are highlighted.

EIS is performed in vitro or in vivo to characterize the electrode—electrolyte or electrode-tissue interface. A schematic representation of the experimental setup for electrochemical impedance spectroscopy and neural stimulation, taken from Dong et al. [21], is presented in Figure 1.

An ultramicroelectrode device, shown in Figure 1(a), has electrodes ranging in diameter from 5 to 50 μm. In vitro EIS is conducted using three-electrode setup as shown in Figure 1(b). Potentiostats provide a small-amplitude sinusoidal potential across a broad frequency range and measure the resulting current. Typical perturbation amplitudes and electrolytes used for in vitro studies are shown in Table 1.

Impedance spectra of neural implants are commonly reported at a single frequency of 1 kHz due to its physiological relevance to neuronal spike timing; however, full spectra of frequency sweeps across 10 mHz to 100 kHz are required to capture time constants associated with double-layer capacitance, charge-transfer resistance, and diffusion effects.

The illustration in Figure 1(c) suggests a typical in vivo application. The in vivo EIS device shown in Figure 1(d) was miniaturized for easy implantation, minimizing tissue damage and inflammation. Most in vivo studies shown in Table 2 use a two-electrode configuration, though some employ a three-electrode setup for electrophysiological analysis. Electrophysiological analyses are performed to measure the electrical activity of neural tissue. The perturbation amplitudes of impedance measurements are selected to maintain linearity while allowing sensitivity to interface changes. In vivo EIS has been conducted in both humans [10] and animals, including rats [31], cats [29], and monkeys [25].

**Figure 2:**
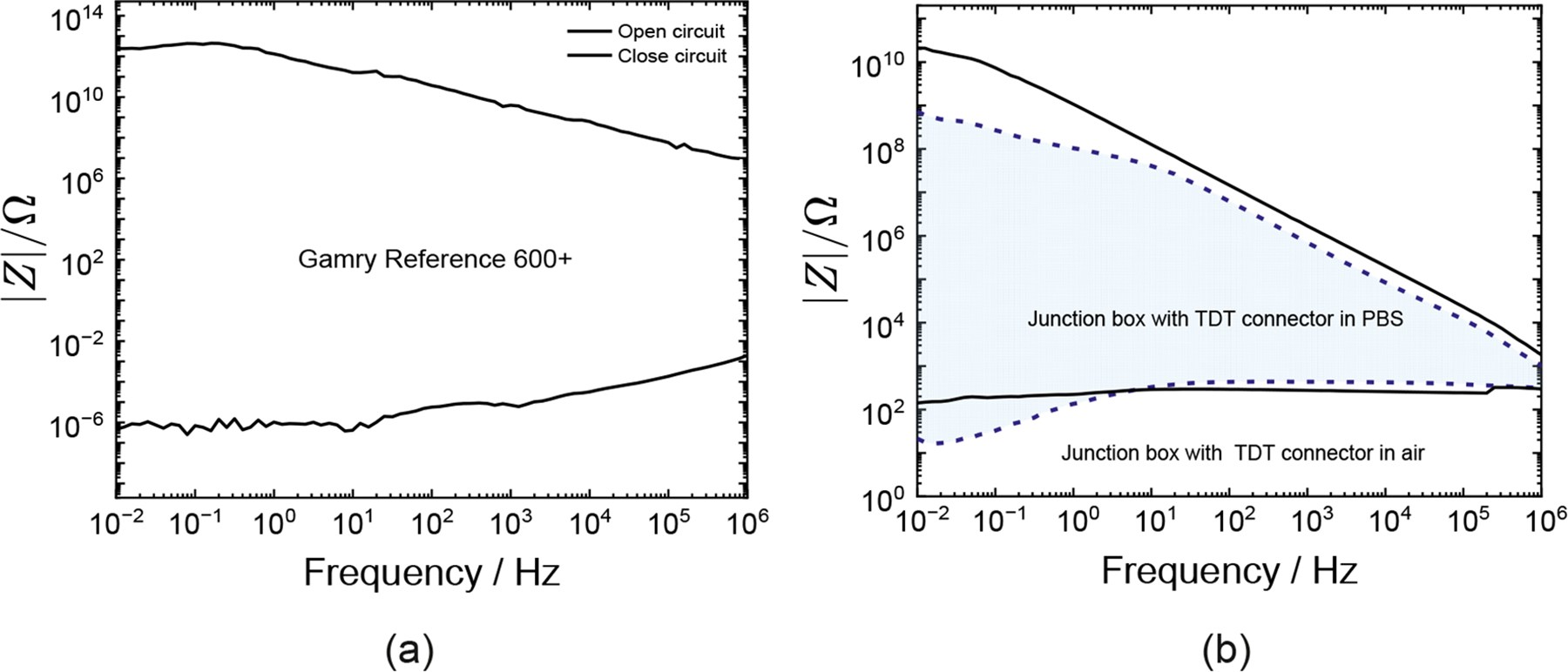
Accuracy contour plots measured in air and PBS at a frequency range from 10 mHz to 1 MHz: (**a**) open-circuit and closed-circuit impedance measurements for the Gamry Reference 600+ potentiostat with 0.6 m cables, and (**b**) accuracy contour plots measured for a Gamry Reference 600+ potentiostat connected to the NeuroNexus device through a custom-built junction box with a TDT ZIF-Clip connector. The boundaries for the maximum and minimum impedance represents open-lead and shorted-lead measurements respectively. Accurate impedance measurements are attainable within the area bounded by upper and lower impedance limits. The useable impedance range was reduced when the custom junction box with a TDT ZIF-Clip connection devices were added to the potentiostat and became much smaller when the entire system was immersed in PBS.

Impedance measurements capture contributions from cables and connectors as well as the electrochemical system of interest. These cables and connectors introduce into the measurement a capacitive or inductive behavior that can confound interpretation of the impedance data. The best approach is to ensure that the measurement does not include the influence of cables and connectors. Accuracy contour plots (ACPs) are used to evaluate the useable impedance and frequency ranges for different instrument setups. Hazelgrove et al. [33] emphasized the importance of accuracy contour plots. Dong et al. [21] plotted accuracy contour plots for the Autolab PGSTAT12 (Metrohm, Utrecht, The Netherlands) by itself and when connected to a neural implant via connection hardware. The impedance and frequency limits of the Gamry Reference 600+ potentiostat alone, and when connected to the ultramicroelectrode array (see Figure 1(e)), are presented in Figure 2. The accuracy contour plots for the potentiostat (Figure 2(a)) were obtained in air using open-lead and shorted-lead configurations. The ultramicroelectrode was tested in open-lead and shorted-lead modes using terminal and looped traces, respectively. Accuracy contour plots for measurements in air and phosphate-buffered saline (PBS) are shown in Figure 2(b). The shaded and unshaded regions represent thresholds in PBS and air, respectively. The useable impedance range decreased when the connection hardware was added to the potentiostat, and was further reduced when the system was immersed in PBS. The results shown in Figure 2 emphasize the importance of assessing the accuracy contour plot using the cables and connectors associated with the system under investigation. It is not enough to report the accuracy contour plot provided by the vendor.

## Interpretation of impedance data

The effective use of EIS requires not just measurements but regression of mathematical models for accurate data interpretation. Circuit-based mathematical models enable interpretation of impedance spectra based on physical and chemical phenomena occurring in the system. Interpretation relies on two principal models: (i) a physicochemical model describing electrode and tissue interactions, and (ii) a measurement model for error structure analysis and assessment of data consistency with Kramers—Kronig relations. The Kramers—Kronig relations [34] were developed for optics and adopted by electrochemists to evaluate the self-consistency of impedance measurements. The measurement model established by Agarwal et al. [35] is a Kramers—Kronig consistent model for analyzing impedance data. Lutz et al. [11] used the measurement model to analyze replicated impedance spectra of SIROF microelectrodes for neural applications. Process models are not unique, and multiple circuits can fit the same impedance data. A review by Vivier and Orazem [36] highlighted a wide range of electrical circuits used to model EIS data.

Constant-phase elements are frequently used to model non-ideal behavior associated with time-constant distributions. The film thickness and effective capacitance can be extracted from CPE parameters. These approaches are evident in neural studies on SIROF microelectrodes [11]. Some neural studies used the Randles circuit to fit impedance spectra. Other studies incorporated Warburg diffusion and resistive films to describe chronic tissue effects.

## Rudimentary use of EIS in neuroscience: a survey and critique

Studies in neural stimulation often apply EIS in a rudimentary way primarily by reporting impedance magnitude and phase without modeling the electrode-tissue interfacial reactions. Researchers measure and report the impedance at 1 kHz because this frequency corresponds to the typical range of neural spikes and stimulation pulses. However, impedance is a frequency-dependent property that reflects both the resistive and capacitive contributions of a system. A single-point measurement at 1 kHz does not capture the electrochemical reactions across a wide frequency spectrum. Hughes et al. [10] measured impedance at 1 kHz to investigate long-term neural stimulation and recording performance of SIROF and platinum electrode arrays, in human sensorimotor cortex over 1500 days. Their results showed that stimulation through SIROF-sensory electrodes maintained lower impedances than platinum-motor electrodes due to material differences and charge injection. However, their study did not account for the faradaic and charge reactions associated with the electrochemical systems. Similarly, Woeppel et al. [32] used impedance measurements at 1 kHz to assess the influence of tissue encapsulation and chronic recording performance of explanted Utah electrode arrays in the human cortex, but did not apply a process model to interpret their impedance spectra. While measuring at a single frequency is fast and convenient for clinical monitoring, it cannot be used to analyze the relevant physicochemical processes such as charge transfer, double-layer capacitance, diffusion, and tissue encapsulation. Advanced interpretation of impedance data across a broad frequency range is necessary to truly separate properties of the electrode from biological tissue responses.

A major gap in the neuroscience literature is the lack of quantitative analysis of error structure [37] and assessment of consistency with Kramers—Kronig relations [35], a test of linearity and stationarity [38] in EIS measurements. Most published neural EIS studies shown in Tables 1 and 2 do not assess the error structure of impedance data. Notably, reviews by Boehler et al. [39] and Schiavone et al. [40] did not discuss the importance of error structure analysis for EIS interpretation. Regression of mathematical models and error weighting are essential to extract statistically significant parameters describing electrode and tissue behavior. Agarwal et al. [35] developed a Kramers-Kronig-consistent measurement model for EIS data regression. The model uses a sum of Voigt circuit elements (*RC* circuits) to represent impedance data without requiring direct Kramers—Kronig integrations. This approach incorporates error analysis and enables precise extraction of electrode-tissue parameters. Bias errors in neural stimulation could arise from instrumentation, biological artifacts, and non-stationary effects. Few studies [18] meet these standards. Lutz et al. [11] used the measurement model program [41] to analyze impedance spectra of SIROF microelectrodes. The maximum number of Voigt elements that yielded statistically significant parameters was employed for regression. They included error analysis to interpret and extract physical parameters of flat substrates and pore walls.

Interpretation of impedance data is often overlooked. Some studies apply electrical circuit models to neural EIS data but fail to integrate error structure into their model. Interpretation models use combinations of resistors, capacitors, constant phase elements (CPEs), and Warburg components to represent the complex processes at the electrode-tissue interface, enabling extraction of physical parameters and improvements in stimulation protocols. O’Sullivan et al. [25] created process models for chronic intracranial electrodes, extracting and interpreting CPE parameters and resistance during long-term implantation. CPE parameters alone are not meaningful and need to be expressed in terms of a capacitance. Many studies report CPE parameters without connecting them to the physical properties such as surface roughness or resistivity distribution. Reviews by Vivier and Orazem [36], Hazelgrove et al. [33], Wang et al. [42] highlight best practices for impedance modeling. Impedance analysis for neural electrodes should include complete impedance spectrum measurement, error structure analysis, assessment with Kramers-Kronig-consistent models, proper regression weighting, and fitting to a meaningful physicochemical interpretation model.

## Major recent developments: improving impedance analysis for neural stimulation

In the past two years, EIS has emerged from being just a monitoring tool to a key method for characterizing neural-stimulation electrodes. Recent brain-stimulation studies use EIS to guide rejuvenation protocols, analyze electrode performance in vitro and under real stimulation conditions, and separate true electrode behavior from measurement artifacts. Latest studies have improved EIS data interpretation using error structure analysis and process models that account for complex interfacial phenomena occurring at the electrode—tissue interface. Current studies perform EIS-based assessment of the long term stability of ultramicroelectrode arrays used in brain-stimulation. The first applications of machine-learning approaches to impedance data in neural-stimulation contexts have emerged.

### Broad frequency range/improved interpretation models

The recent advancements in electrochemical impedance spectroscopy include a broader range of frequency points and improved equivalent-circuit models that incorporate the underlying chemistry and physics of the electrode-tissue interface (ETI).

For example, Lutz et al. [11] used the measurement model to quantify the frequency-dependent error structure of impedance spectra for sputtered iridium oxide film (SIROF) microelectrodes and developed an interpretation model that accounted for the changes in iridium oxidation state and the porous electrode behavior based on the de Levie model. Similarly, Sehlmeyer and colleagues [43] introduced an improved model for cochlear implant electrodes that replaces the simple capacitor in the electrode interface with a non-linear, frequency-dependent polarization capacitance in parallel with a frequency-dependent polarization resistance, alongside a faradaic (charge-transfer) resistance, capturing frequency-dependent behavior missed by conventional models. Also, Sridhar et al. [44] have addressed the inadequacy of linear models to describe the electrode-tissue interface during high-amplitude simulation. When subjected to these large amplitudes, key parameters within equivalent circuit models such as the charge transfer resistance (*R*_ct_) and the double-layer capacitance (*C*_dl_), cease to be constant. Instead, they become dependent on the perturbation amplitude.

### Accuracy contour plots

Parasitic capacitance and instrument limitations have recently become major concerns when interpreting EIS data for modern high-density neural-stimulation electrode arrays. Studies on the impedance spectroscopy of ultramicro-sized SIROFelectrode arrays have shown that the traces, leads, and packaging introduce parasitic capacitances that significantly alter the impedance response of the electrode arrays at high frequency points. As a result, emerging directions emphasize the importance of modeling these parasitic effects and using accuracy contour plots to define the frequency and impedance ranges where reliable impedance spectra can be obtained. Ghazavi et al. [45] quantified how insulation layer thickness, leads and traces in high-density SIROF ultramicroelectrode arrays create parasitic capacitances that alter the high-frequency impedance and charge-injection capacity during pulsing. Their results showed that for the same 20 μm diameter electrode, the array with longer, wider traces and thinner insulation showed lower impedance, reduced charge-injection capacity, and a slightly higher charge-storage capacity than the array with shorter, narrower traces and a thicker insulation layer. Dong et al. [21] demonstrated how the accuracy contour plots can be used to identify the reliable frequency range for high-density ultramicroelectrode arrays.

### Electrode rejuvenation

Studies on the impact of stimulation and rejuvenation on electrode impedance are currently gaining interest following previous work by Otto et al. [17] which showed that rejuvenation voltages improved signal-to-noise ratios and reduced electrode site impedances at 1 kHz. Here, rejuvenation is a brief DC-biasing of the electrode as a quick maintenance step to temporarily restore the performance of chronically implanted microelectrodes. Latest work by O’Sullivan et al. [18,25] also demonstrated that applying brief direct current (DC) or low-frequency alternating current (AC) voltage pulses can reliably and repeatedly reduce impedance in chronically implanted macroelectrodes in non-human primates. This approach, termed “electrical rejuvenation,” is advantageous because it does not require changes to existing electrode designs or the introduction of new materials. The effects, though temporary, can be repeated with brief stimulation sessions, making it a practical solution for maintaining electrode performance over time.

The mechanism/process behind rejuvenation is currently unknown. The performance of a chronically implanted electrode typically degrades, typified by overall amplitude decreases and decreased signal-to-noise ratio of the desired physiological activity.

### Impedance for monitoring

In parallel with these rejuvenation strategies, impedance spectroscopy has emerged as a powerful tool for the real-time monitoring of electrode performance, long-term stability, and tissue response. Maeng et al. [46] illustrated that 10,000 μm^2^ SIROF microelectrodes can safely deliver high-frequency stimulation up to 10 kHz at 250—1000 μC cm^−2^ with minimal electrode degradation, and that stimulation configuration and frequency must be considered when assessing neural stimulation safety. Niaraki et al. [24] used a readily fabricated graphene biosensor to monitor real-time impedance of neural cells in vitro, showcasing high sensitivity, biocompatibility, and the capacity to detect subtle changes in cell—electrode interactions, indicating strong potential for future in vivo applications. Shah and colleagues [47] demonstrated that equivalent circuit analysis of real-time impedance measurements could reveal distinct stages of neural stem cell differentiation, such as changes in cell adhesion and cell—cell contacts, significantly earlier than is possible with conventional microscopic imaging. Park et al. [48] showed that Fourier transform electrochemical impedance spectroscopy (FTEIS) when combined with fast-scan cyclic voltammetry (FSCV) could be used to track electrode surface changes over time. Their findings suggest that an increase in impedance can be caused by a drop in the capacitance, which reflects a loss in the active surface area of the neural electrode.

### Machine learning

Machine learning (ML) is increasingly being used to enhance the analysis and interpretation of impedance of neural stimulation electrodes, addressing challenges in understanding tissue response, electrode degradation, and optimizing performance, among others. A significant challenge in EIS analysis, especially with complex non-linear least squares (CNLS) regression for equivalent circuit model fitting, is the need for accurate initial iterative values and their sensitivity to the results. ML methods, such as Convolutional Neural Networks (CNNs) [49] and Deep Neural Networks (DNNs) [50], have been used to pre-fit EIS spectra, identifying dominating equivalent circuit elements and optimized initial values for regression.

ML is also being used to quantify correlations between impedance and physiological features inferred from model parameters, which can provide important insights into underlying mechanisms. Yi et al. [51] used ML models, including K-nearest Neighbor, Random Forest, Logistic Regression, and Multilayer Perceptron, to predict neurostimulation-induced tissue damage. They showed that the Random Forest algorithm could predict damaging and non-damaging stimulation parameters with an accuracy of 88.3 %, outperforming the Shannon equation which yielded an accuracy of 63.9 %. Straka and colleagues [52] developed Support Vector Machines (SVMs) with a fine Gaussian kernel to categorize impedance spectra into distinct groups (e.g., ““hockeystick,” ““ski-slope,” ““mixed,” and ““outliers”). These categories were then correlated with specific physical changes and abiotic and biotic failure modes, such as tip metal degradation, encapsulation degradation, or wire breakage in the electrode lead. Researchers have also explored machine learning-based surrogate models, such as Multilayer Perceptrons (MLPs), to predict nerve fiber activation under electrical stimulation [53]. It is important to note that while ML can significantly accelerate the analysis of large EIS datasets, the ““black-box” nature of some ML models can make it difficult to generalize derived models or quantify their robustness. Moreover, the performance of ML models is highly dependent on the quality and representativeness of the training data. It is also worth mentioning that equivalent circuit models, and even physicochemical models, of impedance spectroscopy are not unique, and ML applications must be guided by this understanding to suggest physically meaningful solutions.

## Conclusion and outlook

EIS is an indispensable tool for evaluating neural interfaces as it is non-invasive and offers important insights into the chemical and physical processes at electrode—tissue interfaces. Meaningful interpretation of impedance for implanted electrodes requires adherence to best practices that include: measurement validation with the Kramers—Kronig relations, quantification of stochastic error structure, and reporting confidence intervals for estimated parameters and regression statistics. The outlook for impedance spectroscopy for neural interfaces is highly promising. There is a growing and urgent demand for unified standards for neural-electrode impedance measurement, characterization techniques, and data interpretation. Moreover, hybrid approaches that combine physics-based models with machine learning are particularly promising. The ultimate promise lies in embedding impedance monitoring into closed-loop neurostimulation devices. Here, impedance serves as a biomarker for electrode integrity and tissue health, enabling adaptive adjustment of stimulation parameters.

## Figures and Tables

**Table 1:** In Vitro EIS Parameters Applied in Neural Stimulation Studies. All studies referenced employ three electrodes. Details on the electrolytes and electrodes employed can be found in the cited references. The cited references are arranged alphabetically according to the first author’s last name.

Researcher(s)	Electrolyte	Electrode	Electrochemical set-up	Perturbation amplitude	Frequency range	Process model	Error analysis
Abbott et al. [12]	PBS	TiN	CE: Pt, RE: Ag/AgCl	10 mV	1 Hz–100 kHz	No	No
Chakraborty et al. [13]	PBS/MISF	Sputtered RuOx	CE: Large area Pt, RE: Ag/AgCl	10 mV rms	1–100 kHz	No	No
Cogan et al. [22]	PBS	AIROF and PtIr MEs	CE: Large Pt mesh, RE: Ag/AgCl	50 mV	20 Hz–100 Hz	No	No
Dong et al. [21]	PBS	Au coated with SIROF	CE: Pt wire, RE: Ag/AgCl	0.01 V rms	1 Hz–100 kHz	No	No
Fan et al. [9]	PBS	Au and Pt	CE: Bare Pt wire, RE: Ag/AgCl	10 mV rms	1 Hz–100 kHz	No	No
Ghazavi et al. [23]	MISF	SIROF	CE: Large Pt mesh, RE: Ag/AgCl	10 mV rms	1 Hz–100 kHz	No	No
Ghazavi and Cogan [8]	PBS	SIROF	CE: Large Pt mesh, RE: Ag/AgCl	10 mV rms	1 Hz–100 kHz	No	No
Lewis et al. [14]	PBS, 0.01 M	Pt, IrOx, nanoPt, and PEDOT	CE: Not specified, RE: Ag/AgCl	100 mVpp	0.1 Hz–100 kHz	No	No
Lutz et al. [11]	PBS	SIROF	CE: Not specified, RE: Ag/AgCl	10 mV rms	40 mHz–100 kHz	Yes	Yes
Niaraki et al. [24]	PBS	Graphene MEs	CE: Pt wire, RE: Ag/AgCl	10 mV	0.1 Hz–10 kHz	Yes	No
O’Sullivan et al. [25]	PBS/ACSF	DBS and ECoG	CE: Platinum foil, RE: Ag/AgCl	0.01 V	10 Hz–100 kHz	Yes	Yes
Vomero et al. [26]	PS/0.9 % NaCl	Polyimide-based shank with SIROF and PEDOT/PSS coating	CE: Pt, RE: Ag/AgCl	10 mV rms	10–100 kHz	No	No
Woo et al. [27]	PBS	Au nanostructures	CE: Pt	200 mV	100 Hz–10 kHz	Yes	Yes

PBS, Phosphate Buffered Saline; SIROF, Sputtered Iridium Oxide Film; CE, Counter Electrode; RE, Reference Electrode; Pt, Platinum; Ag/AgCl, Silver/Silver Chloride; PtIr, Platinum–Iridium Alloy; DBS, Deep brain stimulation; SRuOx, Sputtered Ruthenium Oxide; MEs, Microelectrodes; IrOx, Iridium Oxide; ASCF, Artificial Cerebrospinal Fluid; MISF, model Interstitial Fluid; PS, physiological saline; PEDOT:PSS, Poly(3,4-ethylenedioxythiophene):Polystyrene Sulfonate; TiN, Titanium Nitride; ECoG, Electrocorticography; PEDOT-CNTs, Poly(3,4-ethylenedioxythiophene)–Carbon Nanotubes Composite; PEDOT-agar, Poly(3,4-ethylenedioxythiophene) embedded in Agar Hydrogel.

**Table 2:** In Vivo EIS Parameters Applied in Neural Stimulation Studies. The cited references are arranged alphabetically according to the first author’s last name.

Researcher(s)	Animal	Electrode	Electrochemical set-up	Perturbation amplitude	Frequency range	Process model	Error analysis
**Three-electrode configuration**							
Abbott et al. [28]	Rat	a-SiC with SIROF sites	CE: Pt wire, RE: Ag/AgCl	10 mV rms	1 Hz–100 kHz	No	No
Cogan et al. [29]	Cat	AIROF	CE: Pt, RE: Ag/AgCl	50 mV	0.01 Hz–100 kHz	No	No
**Two-electrode configuration**							
Black et al. [30]	Rat	SIROF	CE: Stainless steel bone screw	10 mV rms	1 Hz–100 kHz	No	No
Dong et al. [21]	Rat	Au coated with SIROF	RE: Not specified	0.01 V rms	1 Hz–100 kHz	No	No
Hughes et al. [10]	Human	Pt and SIROF MEs	CE: Not specified	10 nA peak-to-peak	1 kHz	No	No
Lewis et al. [14]	Mice	Pt, IrOx, nanoPt, and PEDOT	CE/RE: Ag wire	100 mVpp	1 kHz	No	No
O’Sullivan et al. [18,25]	Monkey	DBS and ECoG	CE: Titanium screw/rod	0.01 V	10 Hz–100 kHz	Yes	Yes
Otto et al. [17]	Rat	Silicon-substrate MEAs with iridium site	CE: Stainless steel bone screw	25 mV rms	100 Hz–10 kHz	Yes	No
Urdaneta et al. [31]	Rat	Silicon-substrate MEAs	CE: Stainless steel bone screw	15 mV peak-to-peak	10 Hz–100 kHz	No	No
Vomero et al. [26]	Rat	Polyimide-based shank with SIROF and PEDOT/PSS coating	CE/RE: Stainless steel bone screw	10 mV rms	10–100 kHz	No	No
Woeppel et al. [32]	Human	Pt, and IrOx MEAs	CE: Not specified	10 nA peak-to-peak	1 kHz	No	No

SIROF, Sputtered Iridium Oxide Film; CE, Counter Electrode; RE, Reference Electrode; Pt, Platinum; AIROF, Activated Iridium Oxide Film; Ag/AgCl, Silver/Silver Chloride; PtIr, Platinum–Iridium Alloy; MEAs; Microelectrode arrays, DBS, Deep-brain stimulation; ECoG, Electrocorticography; Ag, Silver. IrOx, Iridium Oxide; PEDOT:PSS, Poly(3,4-ethylenedioxythiophene):Polystyrene Sulfonate; a-SiC, Amorphous silicon carbide; Au, Gold; PEDOT-CNTs, Poly(3,4-ethylenedioxythiophene)–Carbon Nanotubes Composite; PEDOT-agar,Poly(3,4-ethylenedioxythiophene) embedded in Agar Hydrogel.

## Data Availability

No data was used for the research described in the article.
